# Bridging the Pressure Gap in CO Oxidation

**DOI:** 10.1021/acscatal.1c00806

**Published:** 2021-07-09

**Authors:** Sara Blomberg, Uta Hejral, Mikhail Shipilin, Stefano Albertin, Hanna Karlsson, Christian Hulteberg, Patrick Lömker, Christopher Goodwin, David Degerman, Johan Gustafson, Christoph Schlueter, Anders Nilsson, Edvin Lundgren, Peter Amann

**Affiliations:** † Department of Chemical Engineering, Lund University, Lund 221 00, Sweden; ‡ Department of Physics, Lund University, Lund 221 00, Sweden; § Department of Physics, AlbaNova University Center, Stockholm University, Stockholm 10691, Sweden; ∥ Photon Science, Deutsches Elektronen-Synchrotron DESY, Notkestr. 85, Hamburg 22607, Germany

**Keywords:** Pd(100), CO oxidation, XPS, high pressure, operando

## Abstract

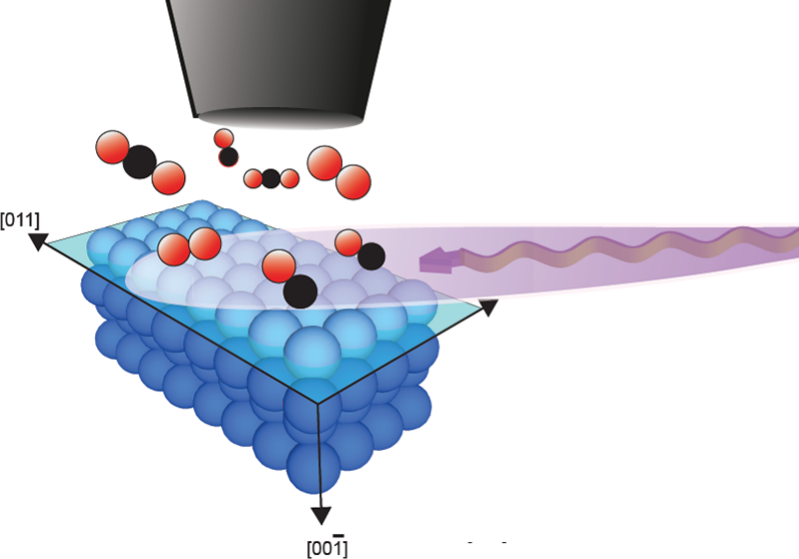

Performing fundamental *operando* catalysis studies under realistic conditions is a key to further develop and increase the efficiency of industrial catalysts. *Operando* X-ray photoelectron spectroscopy (XPS) experiments have been limited to pressures, and the relevance for industrial applications has been questioned. Herein, we report on the CO oxidation experiment on Pd(100) performed at a total pressure of 1 bar using XPS. We investigate the light-off regime and the surface chemical composition at the atomistic level in the highly active phase. Furthermore, the observed gas-phase photoemission peaks of CO_2_, CO, and O_2_ indicate that the kinetics of the reaction during the light-off regime can be followed *operando*, and by studying the reaction rate of the reaction, the activation energy is calculated. The reaction was preceded by an *in situ* oxidation study in 7% O_2_ in He and a total pressure of 70 mbar to confirm the surface sensitivity and assignment of the oxygen-induced photoemission peaks. However, oxygen-induced photoemission peaks were not observed during the reaction studies, but instead, a metallic Pd phase is present in the highly active regime under the conditions applied. The novel XPS setup utilizes hard X-rays to enable high-pressure studies, combined with a grazing incident angle to increase the surface sensitivity of the measurement. Our findings demonstrate the possibilities of achieving chemical information of the catalyst, *operando*, on an atomistic level, under industrially relevant conditions.

## Introduction

Palladium is a well-known catalyst for CO oxidation. The reaction has been studied for several decades, both for industrial applications and to gain a fundamental understanding of the reaction. To achieve fundamental knowledge of the reaction, model systems are often used and studied under well-controlled conditions at low pressures.^1,2^ This is in contrast to the industrial catalyst, which is operated in atmospheric pressure and above. The large difference under applied conditions, often referred to as the pressure gap, has generated an ongoing debate whether the results achieved at low pressures are relevant also for industrial conditions.^3,4^ It has also been recognized that if insights into the reaction mechanism are to be linked to surface structures, the catalyst characterization must be performed under reaction conditions, so-called *operando* studies. To fulfill the criteria of performing *operando* surface-sensitive experiments under realistic conditions, a significant effort has been made to develop experimental setups over the last decades.^5−7^ A well-known model system for fundamental studies of catalytic reactions is CO oxidation using Pd(100) as a catalyst. Several *operando* studies have been performed on the model system where knowledge of the surface structure on an atomistic level has been achieved. By using scanning tunneling microscopy,^8^ polarization modulated infrared reflection absorption spectroscopy,^9^ sum frequency generation,^10^ ambient pressure X-ray photoelectron spectroscopy (AP-XPS),^11^ and surface X-ray diffraction,^12^ the active phase of the Pd(100) surface has been investigated, and both metallic Pd and oxidized Pd have been concluded to be highly active.^13−16^ The active phase is not only dependent on the pressure applied but also on the ratio of CO and O_2_. A general trend is that CO poisoning occurs at low temperatures and high partial pressures of CO resulting in low activity in the reaction. By increasing the temperature, the CO molecules desorb, the light-off for the reaction is reached, and Pd(100) is highly active. For a broad partial pressure range of CO and O_2,_ the surface oxide is reported to be observed after the light-off.^16−18^ The thicker PdO oxide formation has also been observed in the highly active phase when more oxidizing conditions are applied.^12,19^ If the reaction conditions are switched from oxygen-rich to CO rich conditions, a metallic Pd surface is observed.^9^

When CO oxidation *operando* studies of model systems are performed, it has been reported that the geometry of the reactor and the gas flow have a significant impact on gas composition adjacent to the surface.^20−23^ In addition, in an oxygen rich environment, the reaction is most often mass transfer limited (MTL) by CO, immediately after light-off.^9,24^ This is observed as a plateau in the CO_2_ production, and the CO_2_ production is invariant to a further temperature increase. In this regime, the reaction rate is limited by the diffusion of CO molecules that reaches the catalyst. By monitoring the gas phase compositions by means of planar laser-induced fluorescence, the formation of a boundary layer of CO_2_ around the catalytically active surface has been visualized experimentally.^25,26^ The study also reveals that the formation of a boundary layer generates an oxidative environment close to the surface, highlighting the importance of measuring the gas phase close to the catalyst when *operando* studies are performed. To understand the interaction between the gas phase and active surface structure, simultaneous measurements using a combination of several techniques have recently been performed. In these studies, the gas phase, adjacent to the catalytic surface, is being probed simultaneously with the active catalyst surface.^21,27−29^ Using AP-XPS, the gas phase and the surface chemical composition can be probed simultaneously, making AP-XPS a powerful technique for catalysis studies. The advantage of using electron-based techniques, such as XPS, is the surface sensitivity of the measurements enabling the observation of the interaction between the gas molecules and the active site. The short mean free path of the electrons, however, has limited the *operando* AP-XPS studies to tenths of millibar in working pressure.^30,31^ Technical developments utilizing membranes for pressure separation have made high-pressure experiments possible,^32−34^ but the experiments have so far only been on static surfaces under pressure rather than observation of a catalyst during an ongoing reaction. Our previous AP-XPS study of CO oxidation on Pd(100) reports a pressure-dependent measurement using AP-XPS, up to a maximum operating pressure of 1 mbar.^35^ At a total pressure of 1 mbar and 1:1 gas flow ratio of CO/O_2_, a metallic Pd phase is observed immediately after light-off in the highly active phase of the reaction, which is in contradiction to what is observed at higher pressures using other probing techniques.^8,15,36^ To our knowledge, a full range pressure-dependent study of CO oxidation has not been performed previously using a surface-sensitive electron-based technique such as AP-XPS.

A new AP-XPS experimental setup (POLARIS) has been developed at Stockholm University that is currently placed at beamline P22 at the synchrotron PETRA III at Deutsches Elektronen-Synchrotron (DESY) in Hamburg, Germany.^37,38^ The novel setup allows for *operando* experiments performed at 1 bar and above, and industrial-like conditions can be applied. Here, results from an *operando* study of CO oxidation over Pd(100) at total pressures up to 1 bar are presented. In the study, we demonstrate that, by using hard X-ray photoelectron spectroscopy (HAXPES) at the POLARIS setup, it is possible to determine the surface chemical composition at high pressures. Furthermore, the transition from an inactive CO poisoned surface to a highly active phase of Pd(100) is followed *operando*, and knowledge about the surface of Pd(100) during CO oxidation conducted at a high pressure is achieved. The detection of gas-phase peaks in the spectra, originating from CO andCO_2_, is a clear indication of the activity of the Pd(100) surface, making it ideal for detailed studies in the light-off regime. Our study shows that there is no pressure gap at the CO to O_2_ ratio applied, which implies that the results achieved at a low pressure are also relevant under realistic conditions.

## Experimental Methods

The XPS experiment was carried out with the POLARIS^37^ setup positioned at beamline P22 at PETRA III at (DESY) in Hamburg.^38^ The endstation POLARIS utilizes hard X-rays, enabling *in situ* XPS experiments at significantly higher pressures than regular AP-XPS setups. Hard X-rays are used to generate a long inelastic mean free path of the photoelectrons, making regular HAXPES experiments bulk sensitive. At POLARIS, however, the incoming X-rays are impinging on the sample surface at a grazing incident angle, close but below the critical angle of total external reflection, and the measurements can be performed with high surface sensitivity. The Pd(100) single crystal was probed with the incoming photon beam at an incidence angle of 0.6° and a photon energy of 4600 eV, an energy for which the critical angle of Pd corresponds to around 0.8°.^39^ We estimate the probing depth to approximately 10 Å for the Pd metal. To verify a surface sensitivity of the measurements using a grazing incidence angle, we follow the oxide growth *in situ* on the crystal. The surface oxide on Pd(100) has a (√5 × √5)R27° structure and a thickness of one atomic layer, which corresponds to 2.374 Å.^36,40,41^ In previous XPS and AP-XPS studies, we have characterized the (√5 × √5)R27° surface oxide in detail, which can be identified in the Pd 3d_5/2_ and O 1s spectra. It should be noted that the cross section for the s- and p-orbitals do not scale linearly with photon energy, which can be noted in the intensity ratio between the O 1s and Pd 3p_3/2_ core levels when comparing soft and hard X-ray photoemission spectra. In addition, hard X-rays give rise to a slight shift in binding energy due to the recoil effect.^42^

## Results and Discussion

### *In situ* Oxidation at a High Pressure

**Figure 1:**
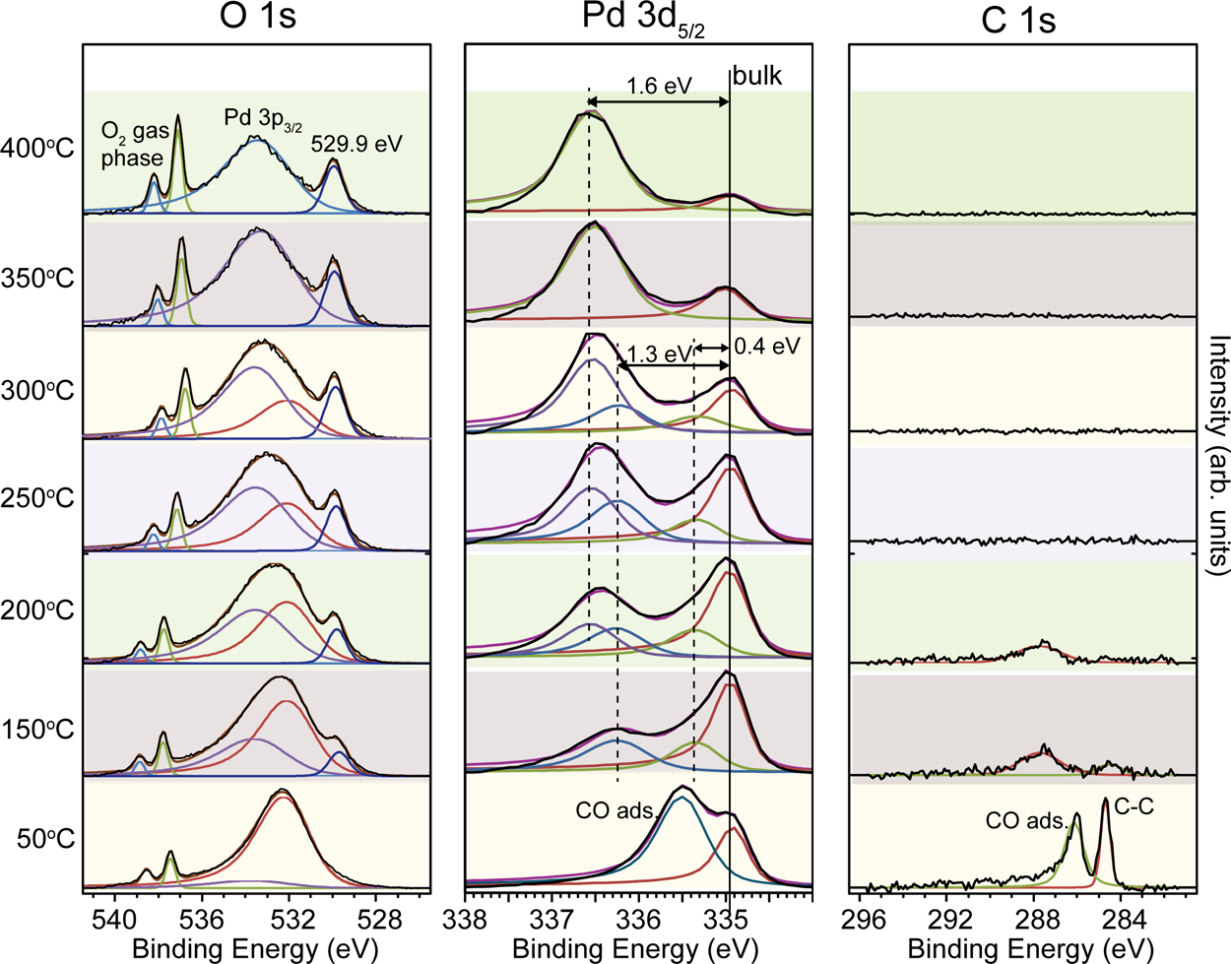
*In situ* oxidation of Pd(100) in the gas mixture of 7% O_2_ in He at 70 mbar total pressure. The temperature is increased in steps of 50 °C between 50 °C and 400 °C. O 1s, Pd 3d_5/2_, and C 1s were measured at each temperature step to follow oxidation.

At the endstation, POLARIS, the surface sensitivity is enhanced by decreasing the incident angle of the X-rays close to the critical angle of the material. To confirm the surface sensitivity of the measurements and to achieve detailed insights into the oxidation process at high pressure, an *in situ* oxidation study of the Pd(100) surface was performed. Based on our previous studies, an oxidative environment is expected at the highly active phase of the CO oxidation reaction and a surface reconstruction may occur due to oxidation. The Pd(100) surface does not reconstruct due to interaction with CO neither thermal-induced reconstruction has been observed.^43,44^ A gas flow of 0.2 L/min O_2_ and 2.84 L/min He, at a total pressure of 70 mbar, was applied in the oxidation experiment. The Pd 3d_5/2_, O 1s, and C 1s spectra were measured, while increasing the temperature from 50 °C to 400 °C (Figure 1). At 50 °C, a bulk component at 335 eV together with a peak shifted by 0.6 eV is observed in the Pd 3d_5/2_ spectrum. The observed core level shift in Pd 3d, together with the peak at 286 eV in C 1s, agrees with previously reported values of adsorbed CO in the bridge site on metallic Pd(100).^45,46^ In O 1s, we observe the two oxygen gas phase peaks centered around 538 eV together with Pd 3p_3/2_. An intensity increase of the gas phase peaks is observed with an increasing temperature, which is due to the thermal expansion of Pd(100) resulting in a smaller distance between the sample and the nozzle.^47^ By increasing the temperature to 150 °C, a significant change in the Pd 3d_5/2_ spectrum is observed. The metal bulk is still the most intense component, but two new components shifted by 0.4 eV and 1.3 eV are observed in the spectrum, which is consistent with the expected peak positions of two- and fourfold coordinated Pd atoms originating in the (√5 × √5)R27° surface oxide.^41^ The assignment of a surface oxide formation is also supported by the new peak observed at 529.9 eV in the O 1s spectrum. The peak at 529.9 eV should contain two components originating from the two different oxygen species in the (√5 × √5)R27° oxide,^41^ but the peaks cannot be resolved and are therefore fitted with a single component in the O 1s spectra. This is also true for the Pd 3p peak, where the two oxidation states of Pd cannot be resolved as clear as in the Pd 3d core level. Therefore, Pd 3p is deconvoluted into two components corresponding to the metallic and oxidized Pd, respectively. The oxidation of the surface implies that the majority of the CO desorb, which is also confirmed by the absent peak in the C 1s spectrum. By increasing the temperature further to 200 °C, the oxide-related peak at 529.9 eV in O 1s is growing, and a new component with a surface core-level shift of 1.6 eV is observed in the Pd 3d_5/2_ spectrum.^45^ This is interpreted as a thicker oxide that is growing on the surface, and we suggest, supported by our previous results, that the thicker oxide grows in islands on the (√5 × √5)R27° oxide.^36^ The conclusion is based on the simultaneous observation of the components assigned to the surface oxide and the thicker oxide in the Pd spectrum. At 250 and 300 °C, the intensity of the metallic bulk component at 335 eV has decreased significantly compared to the oxide-related components, and at 400 °C, it is barely detectable. At 400 °C, a thicker PdO oxide covers the surface, which is also confirmed in the O 1s spectrum, where the ratio between the oxide-related component at 529.9 eV and Pd 3p_3/2_ has decreased significantly. The fitting of Pd 3p is challenging, and we have therefore chosen to fit only the main components in Pd 3p.

Our results demonstrate that the growth of the oxide can be followed *in situ* in the Pd 3d_5/2_ and O 1s spectra at high pressure using grazing incidence HAXPES. The assignment of the components in the spectra is supported by previous *in situ* low-pressure oxidation experiments.^41^ A higher temperature than expected is needed to observe an oxide formation on the Pd surface, which can be explained by the high gas flow that is directed toward the surface, which has a cooling effect on the surface. The temperature is measured with a type N thermocouple at the outer rim of the sample at the front side.

### Operando CO Oxidation at High Pressure

**Figure 2:**
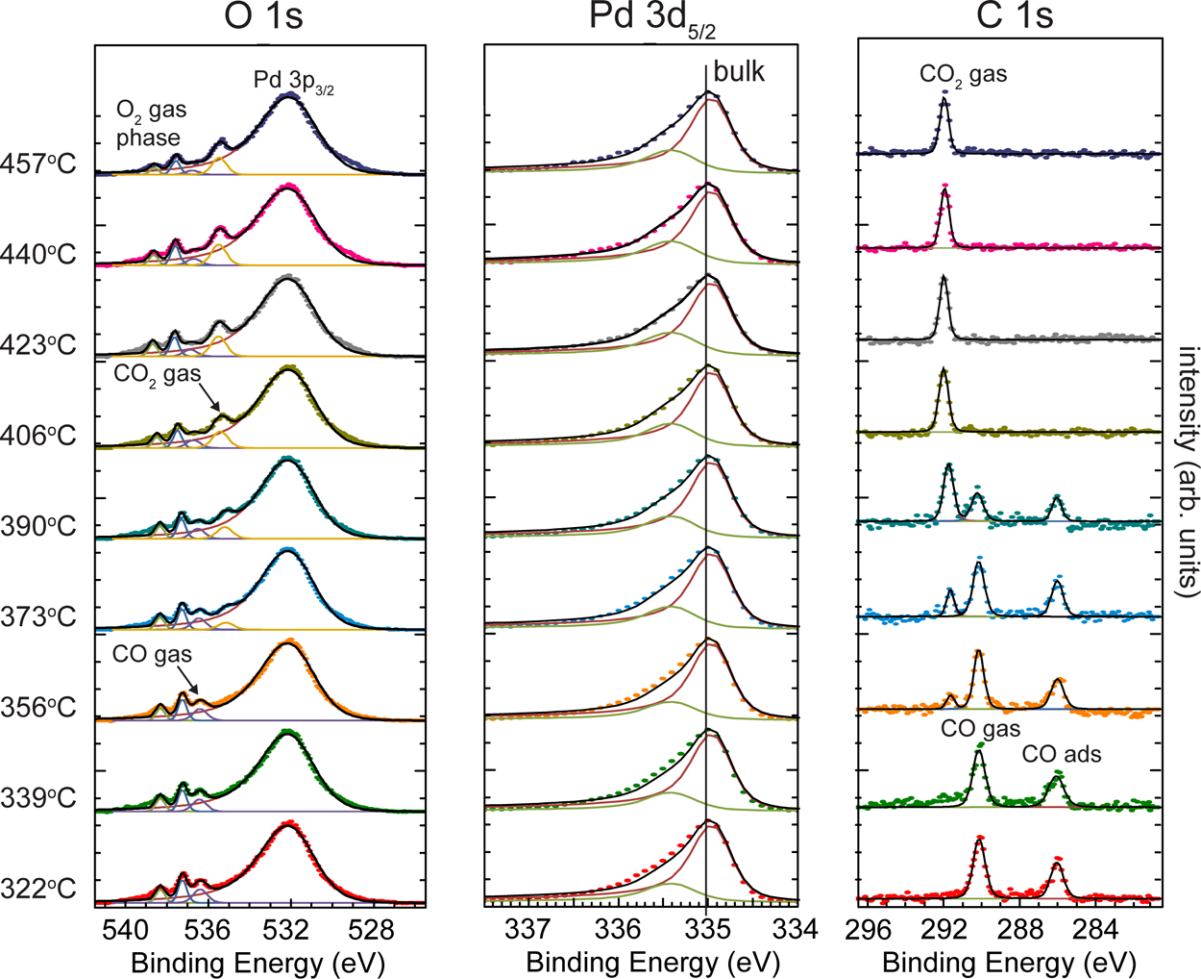
Pd 3d_5/2_, C 1s, and O 1s spectra were measured at a 1:1 gas flow ratio of O_2_ and CO at 100 mbar total pressure. The temperature was continuously increased by 6 °C/min, and the shown temperatures in the figure refer to the temperature when the C 1s spectrum was recorded. At around 355 °C, CO_2_ is detected in the gas-phase in C 1s, which indicates that Pd(100) is active.

In the reaction experiment, the Pd(100) single crystal is used as a model catalyst to gain a more in-depth knowledge of the CO oxidation reaction. In the reported experiment (Figure 2), a total pressure of 100 mbar was applied where an efficient gas transport was achieved by a stagnation flow geometry of the setup using a gas flow of 3.24 L/min with 6% CO and O_2_ each in a gas mixture with He. The Pd 3d_5/2_, O 1s, and C 1s spectra were measured consecutively under these conditions, and the temperature was increased continuously from 320 to 460 °C (6 °C/min), which covers the regime before and after light-off of the reaction. In this study, we ascribe the light-off of the reaction to the moment when the CO_2_ signal can be observed in the C 1s spectrum. Below light-off, a CO poisoned surface is present, which is concluded from a photoemission peak at 286 eV in C 1s, and a related component is observed as a shoulder in the Pd 3d_5/2_ spectrum. The adsorbed CO is also detected in the O 1s spectrum but it overlaps with Pd 3p_3/2_. In O 1s, the CO and O_2_ gas-phase peaks are observed, confirming that a gas mixture of the reactants is present close to the surface. At 320 °C, Pd(100) is inactive and no CO_2_ can be detected, but by increasing the temperature to 355 °C, a peak correlated to gas-phase CO_2_ appears in the C 1s and O 1s spectra. By increasing the temperature further, the CO_2_ gas-phase peak gradually increases, and a corresponding decrease is detected in the CO gas-phase signal. Surprisingly, CO is present in the gas phase and adsorbed at the surface at temperatures up to 405 °C. The results indicate that the reaction is not in the MTL regime immediately after light-off, and the measurement can be performed in the kinetic regime of the reaction. At 405 °C, no CO gas can be detected anymore, and at the same temperature, all CO has desorbed from the surface. Above 405 °C, the reaction reaches a plateau in the CO_2_ production, which is interpreted as the MTL regime. In our oxidation study, we demonstrate that an oxidized Pd(100) surface can be recognized by a photoemission peak at 529.9 eV in O 1s, but in the CO oxidation experiment, no clear evidence of surface oxidation can be observed. A metallic bulk component dominates the Pd3d_5/2_ spectrum, but an additional component shifted toward higher binding energy is also observed. We speculate that the peak is oxygen- or carbon-induced, but the nature behind this peak is not yet understood. A similar component was also observed in our previous AP-XPS results.^35^ At high temperatures, a faint component at 529.9 eV can be observed, which supports our interpretation of chemisorbed oxygen on the surface.

**Figure 3:**
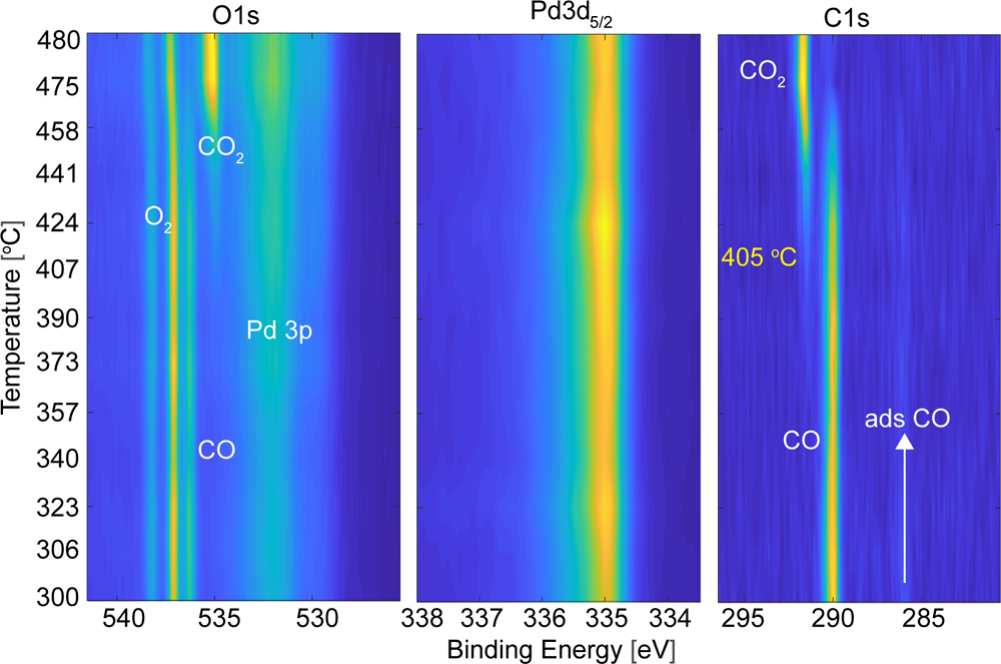
Light-off region for CO oxidation at a total pressure of 1 bar. The O 1s, Pd 3d_5/2_, and C 1s core levels are continuously measured, while the sample temperature is increased by 6 °C/min of the Pd(100) crystal. The CO_2_ gas-phase peak presence is a clear marker of the light-off of the catalyst, which is around 405 °C. The intensity of the spectra is color-coded, ranging from dark blue to yellow, where the latter indicates high intensity.

Characteristic for the POLARIS setup is the high pressure that can be applied. We demonstrate the capacity of the setup by studying the surface structure of Pd(100) within the light-off regime for CO oxidation at a 1:1 gas flow ratio of CO and O_2_ and total pressures up to 1 bar (Figure 3). To ensure to capture the entire light-off regime, we continuously increased the temperature of Pd(100) slowly by 6 °C/min, while measuring the Pd 3d_5/2_, O 1s, and C 1s core levels. The operating pressure of 1 bar is reached by decreasing the working distance between the nozzle and the sample surface to 20 μm. The gas composition of 6% O_2_ and CO in He was used with a total gas flow of 5.18 L/min, which was 0.6 L/min higher compared to the flow used for the measurements at lower pressures. The explored temperature window was between 300 °C to 480 °C, and to keep the working distance, and thereby the pressure, constant over the entire temperature ramp, the sample was manually retracted when required to compensate for the thermal expansion of the crystal.

At 1 bar and 500 mbar (Figure S1 in Supporting Information), the light-off regime features a similar behavior as we observed at 100 mbar total pressure. At 300 °C, the Pd(100) surface is CO poisoned and the sample is inactive, which is confirmed by the absence of CO_2_ in the gas phase. By increasing the temperature of Pd(100), CO starts to desorb, and the light-off is observed at 375 °C at 500 mbar total pressure and at 405 °C at 1 bar. When a maximum temperature of 450 and 480 °C for the study at 500 mbar and 1 bar, respectively, is reached, almost full conversion of CO to CO_2_ is achieved and only a minor CO gas-phase peak is observed in C 1s. During the reaction, the Pd spectra do not change significantly and a metallic surface is observed (Figure S2 in Supporting Information). An oxide formation of the surface would give rise to a peak with a chemical shift of 1.3 or 1.6 eV,^45^ but a peak at these binding energies was not detected at any of the applied conditions ranging from 10^–6^ mbar to 1 bar in total pressure.

### Operando Study in the Kinetic Regime around the Light-Off

**Figure 4:**
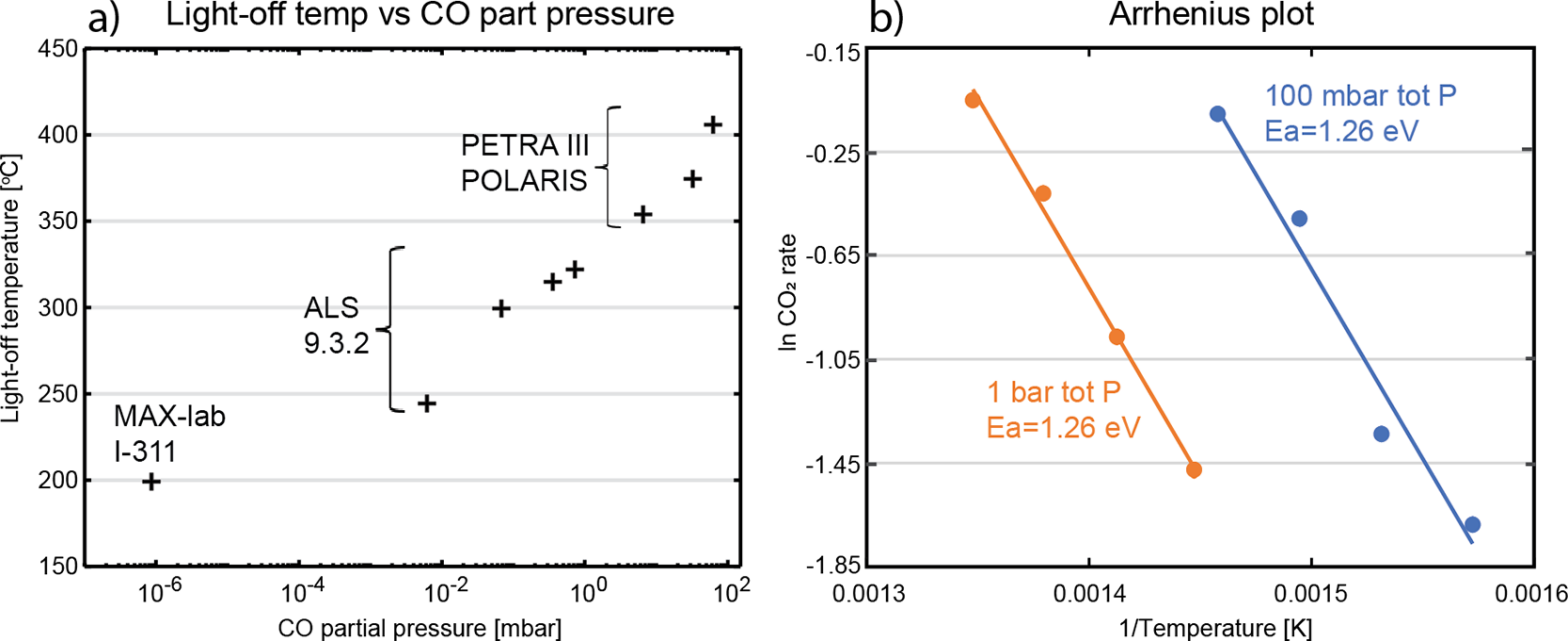
(a) Light-off temperature of the Pd(100) surface as a function of CO partial pressure. The measurements are performed at an increasing total pressure ranging from 10^–6^ mbar to 1 bar in a 1:1 CO to O_2_ gas flow ratio. The synchrotron and beamline where the data are collected are indicated at each data point. (b) Apparent activation energy for 100 mbar and 1 bar is determined from the Arrhenius plot. The CO_2_ formation is extracted from the gas phase peak in the C 1s spectra.

In this study, the light-off temperature of Pd(100) has been investigated at a 1:1 gas flow ratio of CO and O_2_ at pressures between 100 mbar to 1 bar total pressure. This gives additional insights into our previous results on the pressure dependence of the light-off temperature in the same ratio of CO and O_2_ using AP-XPS covering a pressure range from 10^–6^ mbar up to 1 mbar. The results are summarized in Figure 4a, where the light-off temperatures of Pd(100) as a function of the partial pressure of CO are shown. The light-off of the reaction occurs within a temperature window of 200 °C for the whole pressure range, and an exponential dependence of the light-off temperature on pressure is found. The results presented in Figure 4a are generated from experiments conducted at three different endstations and synchrotrons (beamline I311 at MAX-laboratory, Sweden; beamline 9.3.2 at ALS, USA; beamline P22 at PETRA III, Germany), which may introduce uncertainties in the data points relative to each other due to differences in the experimental setup. However, the graph highlights the correlation between the light-off temperature and partial pressure of CO over a wide pressure range, spanning 9 orders of magnitude, establishing AP-XPS as a full pressure range technique.

The light-off regime is investigated in more detail. The simultaneous detection of adsorbed CO and CO_2_ in the gas phase over a wide temperature range at high pressure was surprising to us. A previous AP-XPS experiment with a standard gas flow configuration shows an abrupt change from a CO and O_2_ gas mixture to a CO_2_ and O_2_ mixture present above the surface, immediately after light-off.^35^ In contrast, the POLARIS setup has a high gas flow directed toward the surface, which we speculate suppresses the boundary layer of CO_2_ that is built up around the surface,^20^ and the surface is consequently facing less oxidizing conditions. After light-off, the CO_2_ signal is gradually increasing, while the photoemission peak, originating from the adsorbed CO, decreases, indicating that the CO conversion scales with the number of Pd sites that become available when CO desorbs, which agrees of a reaction pathway according to the Langmuir–Hinshelwood mechanism. To investigate the kinetics during light-off, the effect of the sample temperature on the CO_2_ formation rate was plotted in an Arrhenius form for two total pressures (Figure 4b). During the experiments, no mass spectrometry data were recorded, and the CO_2_ formation rate is extracted from the area of the gas phase peaks in the C 1s spectra for each temperature (Figure S3 in Supporting Information). The slope of the Arrhenius plots gives an apparent activation energy of 1.26 eV (122 kJ/mol K) for both 100 mbar and 1 bar total pressure experiments. Similar activation energy has also been reported in previous studies on Pd(100) and other low index Pd surfaces,^13,48,49^ performed at low pressure and a CO coverage below 0.5 ML on Pd(100),^48,49^ which can be compared to the saturation coverage of 0.75 ML.^46^ In the same study, a decreasing apparent activation energy with increasing CO coverage of the surface is found, which is explained by an increasing repulsion interaction among the adsorbed CO molecules at higher coverage. Our finding of the same apparent activation energy for both, 100 mbar and 1 bar total pressure, indicates that the reaction mechanism has the same rate-limiting step at both low and high total pressures. We speculate that the high gas flow and high temperatures at the *operando* measurements at POLARIS have a non-neglectable impact on the CO coverage and activation energy.

## Conclusions

CO oxidation in a 1:1 gas flow ratio of CO and O_2_ using He as a carrier gas has been studied using Pd(100) as a model catalyst in a total pressure from 100 mbar up to 1 bar. The light-off regime has been studied in detail where the temperature, as well as the surface structure, has been monitored *operando* using AP-XPS. An oxidation study in a mixture of O_2_ and He, with the increasing temperature, was performed to identify the oxidation degree of the Pd surface during CO oxidation. Our results show that we can follow the CO oxidation reaction *operando* by measuring the Pd 3d_5/2_, C 1s and O 1s spectra, while increasing the temperature of the catalyst. The observed Pd 3d_5/2_ core level reveals that metallic Pd is present in the highly active phase of the reaction immediately after light-off at all the applied conditions from low to high pressure. However, in the 100 mbar regime and above, we expected an oxidized surface after light-off due to MTL of CO, as have been reported in previous studies. We speculate that the high gas flow in stagnation flow geometry results in an efficient gas transport of the reactants and products from the surface, which suppresses the boundary layer of CO_2_, and a less oxidative environment is present close to the surface.^20^ The gas-phase peaks detected in the O1s and C1s spectra are used to identify the activity of the catalyst surface, and the apparent activation energy is determined to be 1.26 eV at both 100 mbar and 1 bar total pressure.

Overall, herein we report on the first operando CO oxidation experiments performed using XPS at 1 bar total pressure, which has previously been limited to several orders of magnitude lower pressures. The present results complement our previous *operando* studies using XPS to investigate the light-off regime of Pd(100), where a total pressure range of 9 orders of magnitude has been covered. The study demonstrates the wide pressure range that can be investigated, generating the possibility of performing studies to bridge the so-called pressure gap in several chemical reactions. The temporal resolution of the measurement is sufficient to follow the gradually increased activity, which enables *operando* XPS studies in the kinetic regime of the reaction.

The POLARIS setup has unique possibilities of using photoemission for catalysis research under industrial conditions. The high surface sensitivity of the chemical composition allows for future *operando* studies probing the active site with an atomistic resolution of the catalyst under industrial conditions.

## Supporting Information Available

Cleaning procedure of the sample, CO oxidation at 500 mbar total pressure, C 1s spectra at the light-off regime for 100, 500, and 1000 mbar, Pd 3d_5/2_ spectra measured before light-off, at light-off, and after light-off, and Arrhenius plot data (PDF)
