# Supplementary material for: Bridging the Pressure Gap in CO Oxidation
Source: ACS Catal. 2021 Jul 9;11(15):9128–35. doi: 10.1021/acscatal.1c00806 (PMC8397290; doi:10.1021/acscatal.1c00806)
Supplement: Supplementary file 1 — cs1c00806_si_001.pdf [file cs1c00806_si_001.pdf]

Supporting Information: Bridging the Pressure Gap in CO Oxidation

Sara Blomberg[1], Uta Hejral[2], Mikhail Shipilin[3], Stefano Albertin[2], Hanna Karlsson[1], Christian Hulteberg[1], Patrick Lömker[4], Christopher Goodwin[3], David Degerman[3], Johan Gustafson[2], Christoph Schlueter[4], Anders Nilsson[3], Edvin Lundgren[2] and Peter Amann[3]*

1. Department of Chemical Engineering, Lund University, 221 00 Lund, Sweden

2. Department of Physics, Lund University, 221 00 Lund, Sweden

3. Department of Physics, Stockholm University, AlbaNova University Center, 10691 Stockholm, Sweden

4. Photon Science, Deutsches Elektronen-Synchrotron DESY, Notkestr. 85, 22607 Hamburg, Germany

Corresponding author email address:sara.blomberg@chemeng.lth.se

Cleaning of the sample and gases

The Pd(100) sample was cleaned by sputtering cycles of 5 x 10^{-6} mbar of Ar and annealing to 600 °C. The sample was heated in O_2 to remove carbon contamination on the surface. The annealing temperature could not be increased further, which may affect the order of the clean surface. After experiments where the temperature exceeds 450°C, traces of Si contamination were observed at the surface. However, Si is inactive in the CO oxidation reaction and should not affect the light-off regime significantly.

The gases were dosed via individual mass flow controllers into the chamber. The gases had a purity of 5.0, and an additional carbonyl trap was installed at the CO gas line to avoid contaminations.

CO oxidation at 500 mbar total pressure

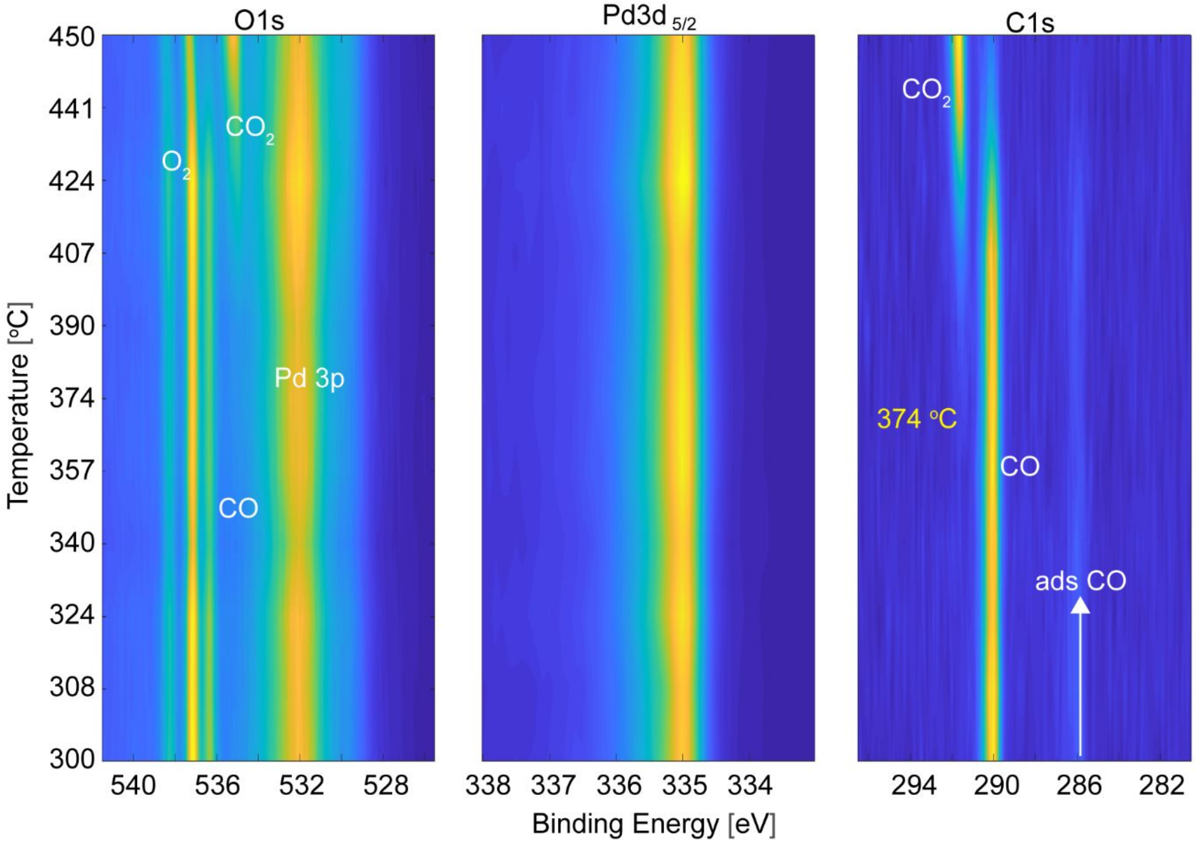

Figure S1. The CO oxidation experiment at 500 mbar total pressure, 0.28 l/min O2, 0.28 l/min CO and 3.98 l/min He, was performed while the temperature was increased from 300 ˚C to 450 ˚C. The working distance between the sample and the aperture of the nozzle was 50 μm. The light-off was observed at 374 ˚C.

Pd 3d$_{5/2}$ spectra measured before light-off, at light-off and after light-off

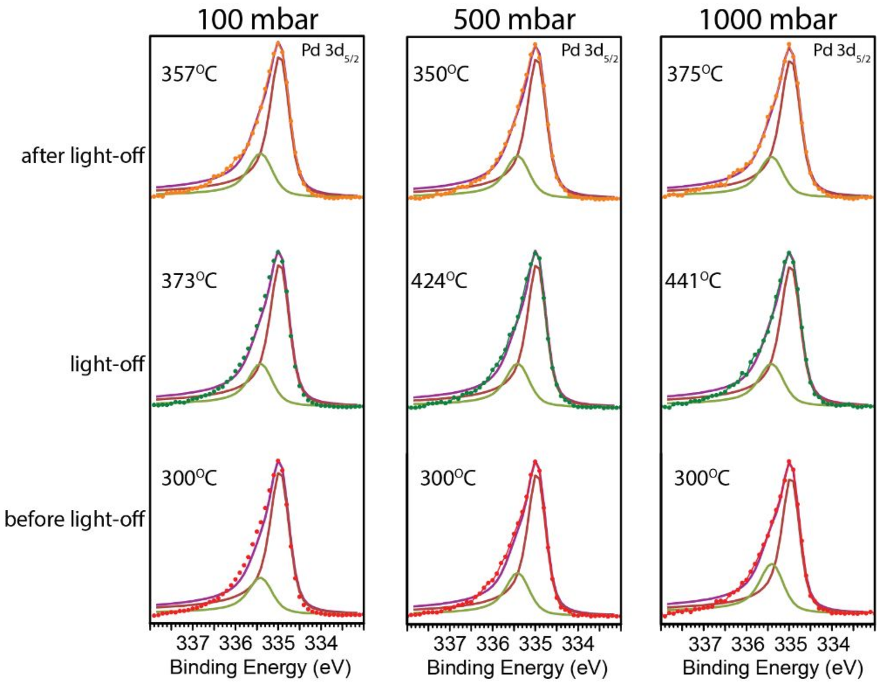

Figure S2. The Pd $3d_{5/2}$ spectra are single scan measurements and observed before light-off, at light-off, and after light-off at three different total pressures. The Pd spectra do not change significantly during the experiments and a metallic Pd phase is observed both before and after light off. The main peak originates from the Pd bulk and before and at the light-off, the component at higher energy is correlated to CO adsorbed on metallic Pd and CO can also be observed in the gas phase in C1s. At high temperatures, after light-off, the component is attributed to chemisorbed O on the metallic surface and the CO gas phase peak in the corresponding C1s spectrum is not observed.

C 1s spectra at light-off regime for 100mbar, 500mbar, 1000mbar

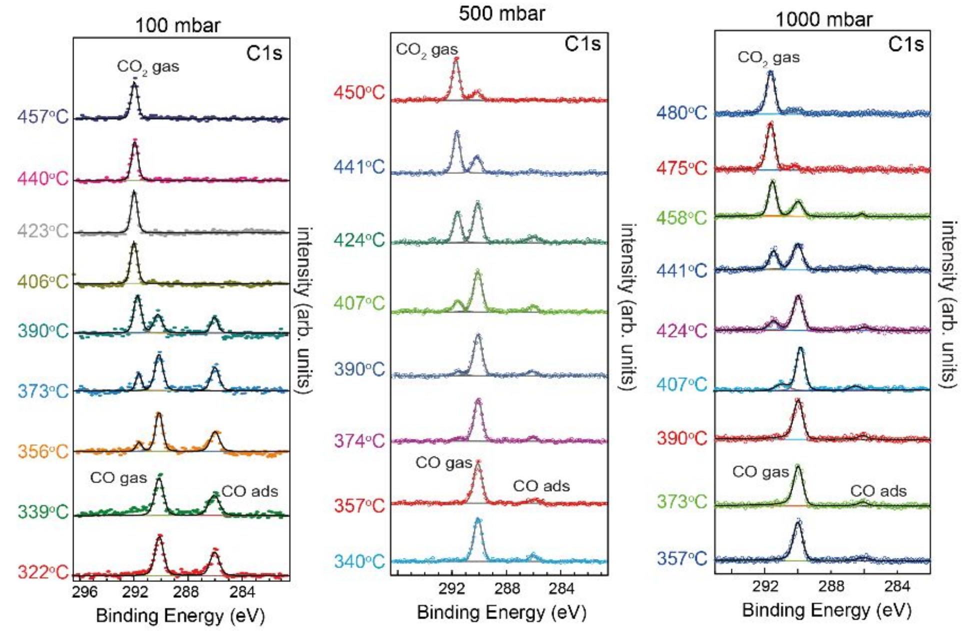

Figure S3. The C 1s spectra are single scan data and were measured during three separate CO oxidation experiments at 100 mbar, 500 mbar, and 1000 mbar total pressure respectively. The temperature of the Pd(100) was increased during each experiment and the activity of the sample was correlated to the CO and CO_2 gas phase peaks in the spectra.

Arrhenius plot

The data for the Arrhenius plot was extracted from the gas phase peaks in the C1s XPS spectra for each temperature according to:

$$\frac{area\ CO_2\ gas\ phase\ peak}{area\ CO + O_2\ gas\ phase\ peaks} = rate\ of\ CO_2$$

Only the data points where an increase in the CO_2 production is observed are included in the Arrhenius plot.
